# Fault Detection in MV Switchgears Through Unsupervised Learning of Temperature Conditions

**DOI:** 10.3390/s25154818

**Published:** 2025-08-05

**Authors:** Grazia Iadarola, Alessandro Mingotti, Virginia Negri, Susanna Spinsante

**Affiliations:** 1 Department of Information Engineering, Polytechnic University of Marche, 60131 Ancona, Italy; s.spinsante@univpm.it; 2 Department of Electrical, Electronic and Information Engineering, University of Bologna, 40136 Bologna, Italy; alessandro.mingotti2@unibo.it (A.M.); virginia.negri2@unibo.it (V.N.)

**Keywords:** fault detection, medium voltage switchgears, distributed measurement system, temperature monitoring, unsupervised learning, clustering algorithms

## Abstract

This paper presents a distributed measurement system intended to effectively monitor the health status of switchgears under varying temperature conditions. In particular, thermocouples are deployed as temperature sensors for the continuous monitoring of a medium-voltage (MV) switchgear. Then, by integrating a low-cost microcontroller unit, the proposed system can implement previously trained unsupervised learning techniques for health status evaluation. This approach enables the early detection of potential faults by identifying anomalous temperature patterns, thus supporting predictive maintenance and extending the lifespan of switchgears. The results show strong clustering performance with low execution times, highlighting the suitability of the method for resource-constrained hardware. Furthermore, onboard temperature processing eliminates the need for data transmission to remote servers, reducing latency and communication overhead while improving system responsiveness. The paper includes a numerical analysis on synthetic data as well as a validation on real measurements. Overall, the presented distributed measurement system offers a scalable and cost-effective solution to enhance the reliability and safety of MV switchgears.

## 1. Introduction

Condition monitoring is a practice introduced to ensure the reliability, efficiency, and safety of electrical power systems by analyzing electrical quantities such as voltage and current, as well as environmental ones, like temperature and humidity [1]. The primary objective is to detect patterns and anomalies that may indicate potential faults or deterioration in the operational health of electric assets. In fact, condition monitoring aims to detect faults, which is crucial in order to maintain stability and prevent failures in power systems.

Advanced techniques exploiting machine learning (ML) have made significant strides in the field of condition monitoring. For example, in [2], fault detection driven by artificial intelligence is developed for electric power components, while the work in [3] is focused on detection and diagnosis in low-voltage distribution networks. Similarly, in [4], a method to detect low-current earth faults in resonant grounded networks is implemented, achieving precise fault localization. Furthermore, a method to improve the performance of distance relays during power swings is proposed in [5], while in [6], mathematical morphology is employed for the reliable detection of high-impedance faults.

These works demonstrate the growing relevance of ML-based techniques in fault detection across various electrical systems. However, most of them rely on supervised learning approaches. Generally speaking, supervised learning and unsupervised learning are two fundamental approaches in ML, each with distinct advantages and drawbacks. Supervised learning excels in tasks where labeled data are abundant, enabling precise and accurate model predictions, particularly for classification and regression problems. However, its reliance on large, annotated datasets can be a significant limitation due to the time and cost involved in data labeling. In contrast, unsupervised learning does not require labeled data, making it ideal for discovering hidden patterns and relationships within data, such as clustering and association tasks. This flexibility comes with a downside: it often yields less accurate results since it lacks the guidance of labeled examples, making the interpretation of its output more challenging. Both approaches are crucial in artificial intelligence, complementing each other depending on the availability of data and the specific problem at hand.

In energy scenarios, for instance, ref. [7] utilized semi-supervised learning for condition monitoring in photovoltaic systems. Similarly, ref. [8] applied supervised ML algorithms to optimize a 200 MW coal-fired boiler with urea injection, achieving cleaner power generation and reducing emissions. In [9], transformer-based self-supervised learning methods are developed to improve performance in high-voltage fault diagnosis, even with limited labeled data, demonstrating the adaptability of supervised approaches in practical applications. On the other hand, unsupervised learning, often combined with supervised methods, provides robust solutions in scenarios with limited labeled data. In [10], a hybrid approach of unsupervised and supervised learning is employed to forecast the absolute open flow potential for shale gas reservoirs. In [11], a global solar radiation forecasting system is developed using combined learning models, improving the accuracy of solar energy predictions. In [12], unsupervised learning for solving AC optimal power flows is explored, offering innovative designs and analysis for power system optimization. Additionally, ref. [13] proposed a new unsupervised method for voltage-sag source detection, contributing to more reliable and sustainable energy grids.

Despite these advancements, there is a notable gap in applying unsupervised learning techniques for real-time fault location in medium-voltage (MV) switchgears, especially under practical constraints such as limited labeled data and low computational resources in field devices. Most existing studies assume the availability of extensive labeled datasets and use computationally intensive models that are not feasible for deployment on microcontroller-based platforms. This highlights the need for solutions that can operate efficiently in constrained environments while still providing reliable fault detection and localization.

Therefore, this paper aims to develop a microcontroller-based condition monitoring system enhanced by unsupervised learning to implement fault location in MV switchgears. The choice of an unsupervised learning approach is due to the typical lack of significant information from electric assets, especially in the MV domain, which makes a supervised approach not always practical. The microcontroller-based solution is chosen for its cost-effectiveness, reliability, and scalability, enabling it to cover a wide portion of the electric grid without significantly impacting the economic operation of the distribution system.

In order to evaluate the proposed solution, both synthetic and experimental datasets are used. The synthetic datasets include uncertainty-augmented temperature readings, allowing us to test the robustness of the clustering algorithms under noisy conditions. The experimental data are acquired from a real distribution monitoring system (DMS), using two thermocouples to measure temperature variations under different operating conditions of a commercial MV switchgear.

All the above constitutes the added value of the work, which is further stressed in the specific sections of the paper. Section 2 introduces the motivation of the work. The proposed approach is described in Section 3, highlighting the capabilities of the adopted solution. Then, Section 4 includes the experimental analysis. Finally, the summary of the achievements is given in Section 5.

## 2. Motivation

In this section, the motivation behind the work is detailed and contextualized. MV switchgears face several operational challenges that can impact their reliability, safety, and lifespan [14]. These challenges often arise from a combination of electrical, mechanical, environmental, and even human factors. Of course, electrical issues are a primary concern for MV switchgears: overloading, overheating, short circuits, up to catastrophic failures, may happen for a number of conditions. Among them, it is of relevance to consider insulation failures. The insulation system, which is critical for preventing electrical faults, may degrade over time due to moisture, temperature fluctuations, and contaminants, leading to short circuits and, in severe cases, arc flash hazards and fires [15]. Overheating may happen because of overcurrent conditions, when the current flowing through the equipment exceeds its rated capacity, or unstable power quality generates voltage fluctuations and harmonic issues. A localized dielectric breakdown of a small portion of the insulation system under high-voltage stress generates a partial discharge. Partial discharges, caused by defects within the insulation material (such as voids, cracks, or contaminants), often start small and undetectable but can progressively damage insulation, leading to flashover and equipment failure [16].

Mechanical issues are a significant contributor to switchgear problems, sometimes accounting for a large percentage of failures. Faulty or loose electrical connections increase resistance, leading to overheating, arcing, and damage to components such as circuit breakers and busbars [17]. The same components, including motion mechanisms, can deteriorate over time due to normal wear, mechanical stress, and environmental factors. Among the latter ones, it is worth mentioning the following: vibrations from nearby machinery or seismic activity, which can loosen connections and cause mechanical stress on switchgear components, contributing to failures; the accumulation of dust, dirt, and airborne pollutants (such as industrial chemicals, salt from coastal areas, or exhaust fumes) which can degrade insulation, cause tracking, and lead to the corrosion of metal parts, thus impairing performance and increasing the risk of faults, as well as high humidity and moisture causing condensation and extreme temperatures. Both excessively high and low temperatures can affect switchgears. High temperatures can cause overheating and accelerate insulation degradation, while low temperatures can impact the performance of mechanical components and control systems [18]. Temperature acts as a dominant degradation factor, significantly shortening insulation life and accelerating the aging process of mechanical and electrical parts. This inherent vulnerability to thermal stress makes temperature monitoring and management a critical focus for both the design and ongoing maintenance of switchgears. From an operational standpoint, this means that maintaining temperatures within specified limits is not merely a compliance issue but a fundamental requirement for ensuring the long-term operational efficiency and safety of the equipment.

Documents from organizations such as the IEEE Standards Association [19] often include sections related to temperature rise limits and the importance of thermal monitoring for ensuring equipment reliability and safety. Industry best practices, such as those published by the International Electrical Testing Association [20], provide guidelines for interpreting thermal images and determining maintenance actions based on temperature differentials. Continuous monitoring, on the other hand, which accounts not only for temperature but also other influencing quantities, directly contributes to extending the operational lifespan of the switchgear, thereby reducing the frequency of costly repairs and unplanned downtime.

Condition monitoring can be considered a good solution to prevent and locate faults. To this purpose, local monitoring systems or, more generally, DMSs are employed. In the former category of solutions, it is possible to mention infrared thermography-based diagnostics based on the processing of thermal images [21,22], wireless monitoring methods used to identify thermal stresses in MV switchgear cabinets [23], and more general fault detection solutions in which the classification accuracy of arcing and tracking is improved by considering thermal anomalies.

Regarding DMSs, a method is presented in [24] for distributed voltage angle and frequency droop control in microgrids. A multi-area distribution state estimation approach with nodal redundancy is introduced in [25]. The work [26] proposes, instead, an impedance-based fault location method using both synchronized and unsynchronized current measurements. In [27], a phase locked loop acquisition system performance is evaluated under off-nominal conditions, demonstrating its potential for cost-effective and reliable power system monitoring.

Several studies have explored many aspects for improving the reliability of switchgears. In [28], the temperature rise of gas insulated switchgear disconnectors is investigated with finite element analysis. In [29], a partial discharge detection sensor for gas insulated switchgears is developed, integrating optical and ultra-high-frequency methods. In some cases, the research is also focused on online monitoring systems for multi-state quantities characteristic of high-voltage switchgears [30,31] or on monitoring systems for switchgears using fiber-optic technology [32]. The literature reports several examples of DMSs applied to electrical equipment and operated within networks of monitoring nodes by using different communication technologies, either wireless or wired ones, such as in [33,34,35]. Based on the existing body of knowledge, scaling the proposed approach to a network of DMSs located inside different switchgears of a substation would exploit proper networking, synchronization and data aggregation strategies already available as different options, according to each specific application case. Overall, the current literature does not focus on the exploitation of a DMS for diagnostic purposes that benefit the predictive maintenance of MV switchgears. Furthermore, there are no studies that link temperature, ML, and the status of MV switchgears.

In this paper, the DMS is integrated into the MV switchgear cabinet to enable condition monitoring and early fault detection. These faults are linked to temperature variations caused by incorrect mechanical fastenings within the switchgear [36]. In order to improve diagnostic capabilities, the DMS is enhanced with unsupervised learning running on a resource-constrained microcontroller. Compared to a previous study [37], which employs supervised learning trained on labeled temperature measurements, the present work introduces several key advances. Firstly, the temperature monitoring strategy is based on a different configuration of sensor deployment at measurement sites, providing a more targeted health status of the switchgear. The main novelty lies in employing unlabeled temperature measurements, since labeled data are often unavailable or impractical to obtain in real-world scenarios, making the proposed approach particularly suitable for situations where the health status of the switchgear is unknown. Thus, clustering techniques are explored to determine whether underlying fault categories can be inferred in the absence of labels: the approach proposed here can potentially be adapted to a broader variety of operating conditions. Anyway, for evaluation purposes, the performance of clustering outcomes is also analyzed in terms of labeled datasets. Finally, the unsupervised inference pipeline has been deployed on a low-power microcontroller embedded within the DMS. This integration supports fully real-time fault detection without requiring data transmission to remote servers, making it suitable for edge-level applications in industrial settings. The validation presented here, based on a cost-effective and scalable solution for condition monitoring, demonstrates the feasibility and efficiency of unsupervised on-board learning for fault detection in MV switchgears by autonomous DMSs.

## 3. Proposed Approach

**Figure 1:**
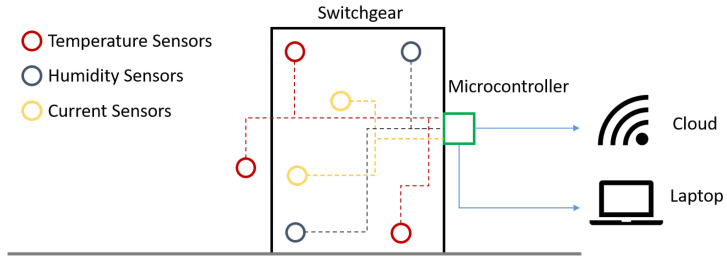
Conceptual scheme of the proposed approach for monitoring switchgears.

This section is dedicated to the proposed approach for monitoring switchgears, describing the in-field implementation of DMSs, together with microcontroller-based unsupervised learning. The proposed approach is schematized in Figure 1. It includes the switchgear, the microcontroller and, by way of illustration, some current, temperature, and humidity sensors. Specifically, the adopted implementation only exploits temperature measurements because, after exploratory testing, they were found to be the most significant when it comes to the health estimation. Temperature measurements are processed by a microcontroller embedded in the DMS, which performs real-time health status inference directly onboard. The processed information can then be transmitted to a local computer or cloud-based infrastructure for further analysis or storage. In any case, the proposed approach is devised to operate regardless of centralized servers, leveraging edge-level processing for efficient and autonomous fault detection. Furthermore, the proposed approach aims at providing a generic but efficient measurement setup that can be customized and even improved by any final user (depending on the characteristics of their application). In fact, every application has several constraints like costs, immunity, dimensions, channels, etc.

This edge-level architecture offers several advantages: it minimizes latency, avoids dependence on communication infrastructure, and reduces bandwidth requirements. Most importantly, it enables local and immediate decision making, which is crucial in time-sensitive applications such as fault detection. In this work, real-time classification performance was practically evaluated by running the clustering inference onboard the microcontroller using actual temperature measurements. The response time was verified to be compatible with real-time diagnostic needs, demonstrating the feasibility of the proposed implementation in realistic operating conditions.

The experimental setup for the case study consists of an MV switchgear and a data acquisition system to record temperature values. The DMS consists of SR30KX K-type thermocouples (TC Ltd., Uxbridge, UK), to locate inside the MV switchgear and connect to a data acquisition system, i.e., a cDAQ-9178 CompactDAQ (National Instruments (NI), Austin, TX, USA) chassis. The cDAQ-9178 CompactDAQ chassis is supplemented with NI-9214 C Series Temperature Input Modules (National Instruments (NI), Austin, TX, USA), which convert temperature-dependent voltage provided by thermocouples in temperature values [38]. As displayed in Figure 1, the DMS can be equipped also with thermocouples outside the switchboard, Rogowski coils to measure the current on the three-phase system, and humidity sensors, enabling comprehensive monitoring of the MV switchgear.

The DMS system employs an 33 BLE Sense Rev 2 (Arduino Nano, Monza, Italy), which is a compact and cheap board that includes Bluetooth Low Energy connectivity, making it ideal for IoT prototyping and experimentation. The board is powered by the nRF52840 microcontroller (Nordic Semiconductor, Trondheim, Norway), which operates at 3.3 V and runs at a clock speed of 64 MHz. The board features 14 digital input/output pins, 8 analog input pins and an I2C bus.

### 3.1. Condition Monitoring of MV Switchgear

The switchgear employed for condition monitoring is an Imesa MINIVER/C MV switchgear (I.M.E.S.A. S.p.A., Jesi, Italy), i.e., a metal-enclosed switchboard suitable for voltages exceeding 1 kV, and up to and including 52 kV, following International Standard IEC 62271-200 [39]. It is made up of four compartments: a busbar compartment (a), a line compartment (b), a circuit breaker compartment (c), and a low-voltage compartment (d). The busbar compartment contains two copper busbars, which are linked by a joint and connected to the fixed contact of a higher interrupting device envelope. Thus, in the line compartment, the current is reduced by a factor of 750 through a Jaes WATTSUD IWR10K (Wattsud L.e.p. S.p.A., Casavatore, Italy) current transformer working at a frequency of 50 Hz. The line compartment is also designated for hosting the lines that connect the power cables, arranged in the rear part, to the fixed contact of a lower interrupting device envelope. Finally, the switchgear is endowed with an interrupting device and low-voltage equipment, placed, respectively, in the circuit breaker compartment (c) and in the low-voltage compartment (d). The switchgear is supplied by a current of 630 A.

**Figure 2:**
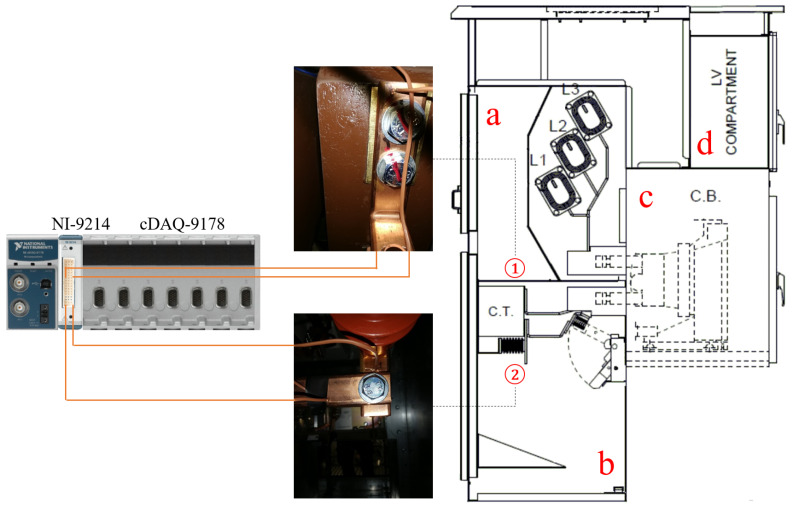
Experimental setup to monitor site (1)—upper contact with the current transformer (CT)—and site (2)—connection to power cables—inside the MV switchgear composed by busbar compartment (a), line compartment (b), circuit breaker compartment (c), and low-voltage compartment (d).

In the current work, variations in temperature were observed in response to variable fastening on the current transformer (CT) upper contact while fixing correct operating conditions for all the other fastenings. For this reason, two sites were chosen to monitor the temperature values: (1) upper contact with the current transformer, and (2) connection to the power cables. Figure 2 illustrates the complete adopted experimental setup. In particular, since one NI-9214 Temperature Input Module can host up to sixteen thermocouple input channels, it can acquire simultaneously the temperature values from both thermocouples, occupying a single slot among the eight available of the cDAQ-9178 CompactDAQ chassis.

**Figure 3:**
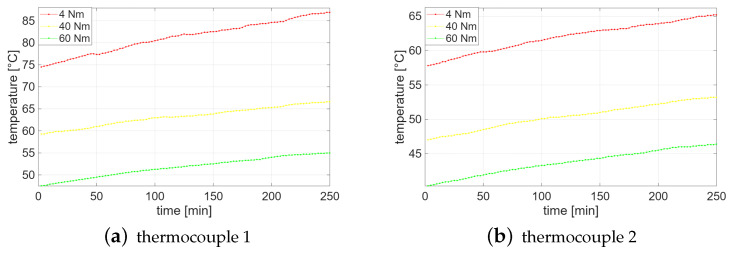
Increasing trend of temperature acquired on site (1) and site (2) through both thermocouples by fastening the upper contact with the current transformer by a torque of 60 Nm, 40 Nm and 4 Nm.

**Figure 4:**
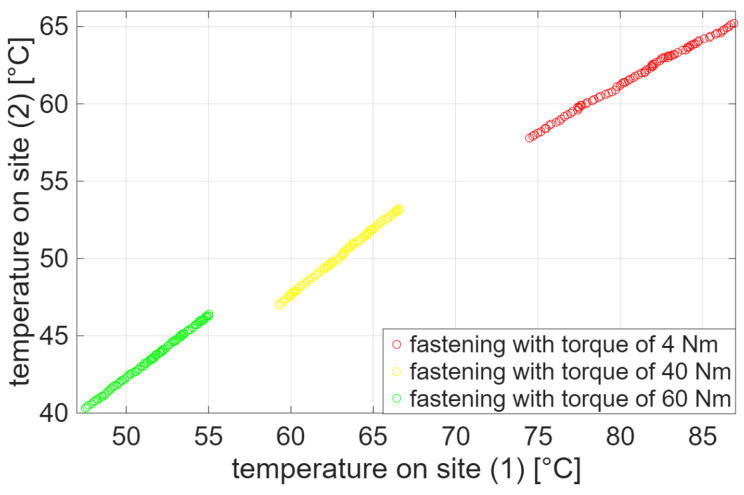
Scatter plots of temperature values acquired on site (1) and site (2) through both thermocouples by fastening the upper contact with the current transformer by a torque of 60 Nm, 40 Nm and 4 Nm.

Condition monitoring was realized in three separate sessions. The M10 screws ensured the power cable connection was fixed at a standard operational torque of 45 Nm, which did not change during experiments, while the torque applied to the upper contact with the current transformer was changed at each session. During the first session, the upper contact with the current transformer was fastened with a torque of 60 Nm to monitor temperature increase under the correct operating conditions of the MV switchgear. In the two following sessions, the fastening on the current transformer was gradually loosened. Specifically, during the second session, the current transformer was slightly fastened with a torque of 40 Nm. Lastly, during the third session, the current transformer was intentionally fastened with a torque of 4 Nm in order to monitor temperature increase in faulty conditions of the MV switchgear. For each value of fastening, the MV switchgear was switched on 3 h and 30 min before starting the acquisition. Then, 100 temperature values were sampled by each thermocouple for each fastening: one every 2 min and 30 s since the beginning of the acquisition session. The complete trend of temperature increase measured through both thermocouples during the three sessions is shown in Figure 3. Figure 4 instead shows the scatter plots of temperature values acquired on site (1) and site (2). Specifically, the scatter plots highlight that such temperature measurements are well suitable to clustering analysis. Finally, Table 1 details the initial and final temperature values for each acquisition session.

Only temperature measurements from thermocouples in site (1) and site (2) of Figure 2 were used to discriminate the different conditions of the MV switchgear. This choice was supported by analyzing the contribution of other sensors, which proved to be not relevant. For example, monitoring the temperature of internal air or other specific sites, as well as the current on the three-phase system, the variations dictated by fastening were shown to be minimal for the three sessions. Indeed, as displayed also in previous works [36,37], no substantial temperature variation can be observed when monitoring the fixed contact with the higher interrupting device envelope and the joint between the busbars in the busbar compartment, or the fixed contact with the lower interrupting device envelope in the line compartment, or even the lower and upper tulip of the circuit breaker. Similarly, the current of the three-phase system was shown to be stable for the three sessions. Its measured values are on average, in fact, equal to 633.5 A, 633.6 A and 635.9 A, respectively.

### 3.2. Unsupervised Learning

In modern power systems, clustering algorithms are crucial for automated decision-making processes when handling large datasets. Among unsupervised learning methods, the *K*-means algorithm is widely used due to its efficiency and simplicity of implementation [40]. It is well suited for large datasets, as it is scalable, managing increasing volumes of data without a significant loss in performance. In this work, *K*-means was selected specifically for its balance between computational efficiency and adaptability in the context of large-scale power system data. The algorithm works by partitioning data into *k* predefined number of clusters, assigning each data sample to the nearest cluster center, called a centroid, and updating the centroids iteratively.

The *K*-means algorithm has been applied to temperature measurements, both real and synthetically generated, collected from switchgear compartments under different fastening conditions. The initialization was performed using the *K*-means++ method to ensure well-separated starting centroids.

Initially, a centroid of cluster *k*, $\mu_{k}$, is randomly selected from the data points. The distance between a data point $x_{i}$ and the centroid $\mu_{k}$ of cluster *k* is typically calculated using the Euclidean distance:

(1) $$d\left(x_{i},\mu_{k}\right)=∥x_{i}-\mu_{k}∥.$$

After all data points are assigned to clusters, the centroids are updated. The new centroid for cluster *k* is denoted as $\nu_{k}$ and is computed as the mean of all points assigned to that cluster:

(2) $$\nu_{k}=\frac{1}{N_{k}}\sum_{x_{i}\in C_{k}}x_{i},$$

where $N_{k}$ is the number of points in cluster *k*, and $C_{k}$ is the set of points assigned to cluster *k*. This assignment and update process is repeated iteratively until the centroids ($\nu_{k}$) no longer change significantly, or a maximum number of iterations is reached [41]. The *K*-means algorithm seeks to minimize the cost function, which is defined as

(3) $$J=\sum_{k=1}^{K}\sum_{x_{i}\in C_{k}}{∥x_{i}-\nu_{k}∥}^{2}$$

where *J* represents the total within-cluster variance, *K* is the number of clusters, and $C_{k}$ denotes the set of points assigned to cluster *k*.

Despite its advantages, the *K*-means algorithm has notable drawbacks. It performs well under the assumption that clusters are spherical, equally sized, and well separated in the feature space, which often does not hold in real-world scenarios. When clusters have non-convex or elongated shapes, or significantly different densities, *K*-means tends to produce inaccurate assignments due to its reliance on Euclidean distance and centroid-based partitioning. Moreover, the algorithm is highly sensitive to outliers, as a single distant data point can disproportionately influence the position of the centroids, thus degrading the quality of the clustering.

**Figure 5:**
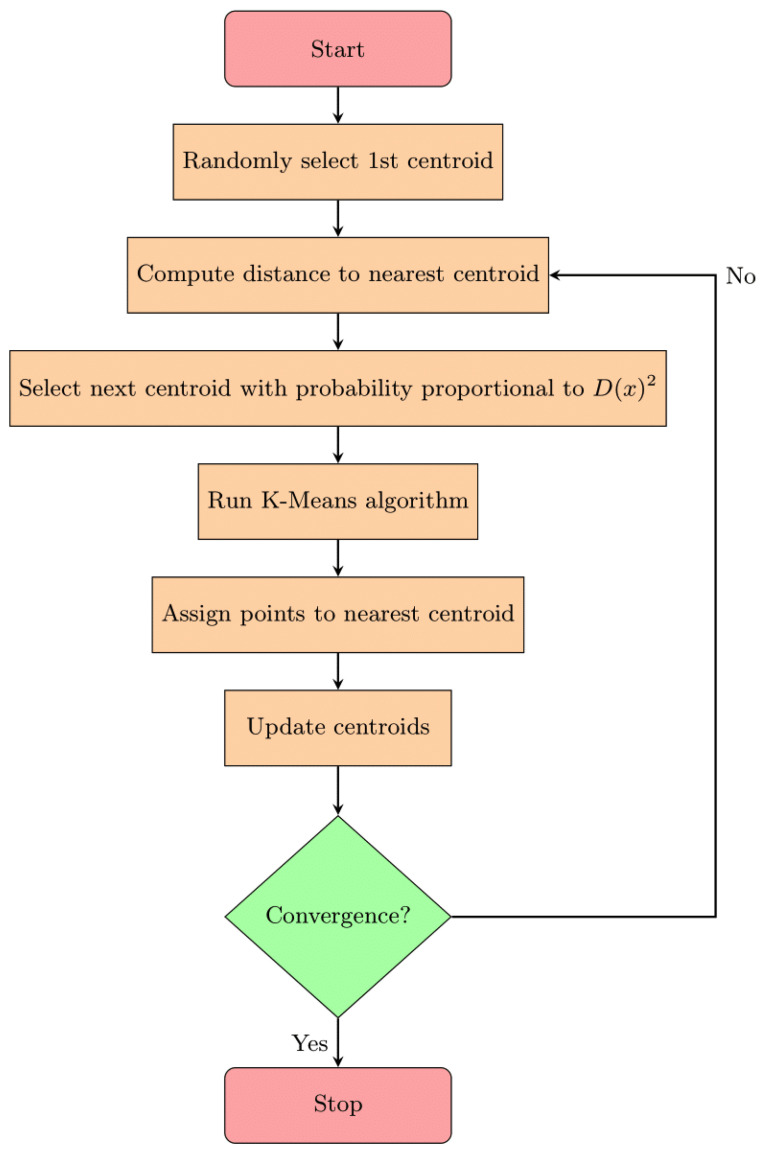
Flowchart of the *K*-means++ algorithm.

Another critical limitation is its sensitivity to the initial selection of centroids ($\mu_{k}$), which can lead to suboptimal solutions by converging to local minima rather than finding the global optimum solution [42]. To address this, the *K*-means++ initialization is introduced. Specifically, instead of random centroid selection, *K*-means++ starts with one random centroid and then, for each subsequent centroid, the distance D(x) from each data point *x* to the nearest already chosen centroid is computed. The distance is given by

(4) $$D\left(x\right)=\min_{j}{∥x-\mu_{j}∥}^{2}$$

where $\mu_{j}$ denotes the previously selected centroid. The probability p(x) of selecting a point *x* as the next centroid is proportional to $D{\left(x\right)}^{2}$, ensuring that points farther from the existing centroids are more likely to be chosen:

(5) $$p\left(x\right)=\frac{D{\left(x\right)}^{2}}{\sum_{x^{\prime }\in X}D{\left(x^{\prime }\right)}^{2}}$$

where *X* represents the set of all data points. The process is repeated until all *K* centroids are chosen. Figure 5 represents the flowchart of the *K*-means algorithm with the *K*-means++ initialization. This approach ensures that centroids are evenly distributed and well separated from each other [43].

Given the above-mentioned limitations, spectral clustering was implemented as well in order to compare its performance with *K*-means performance. Spectral clustering does not rely on centroid-based assumptions and is better suited to identify arbitrarily shaped clusters, making it a valuable benchmark for assessing the robustness of the results obtained with *K*-means.

### 3.3. Implementation on Microcontroller

**Figure 6:**
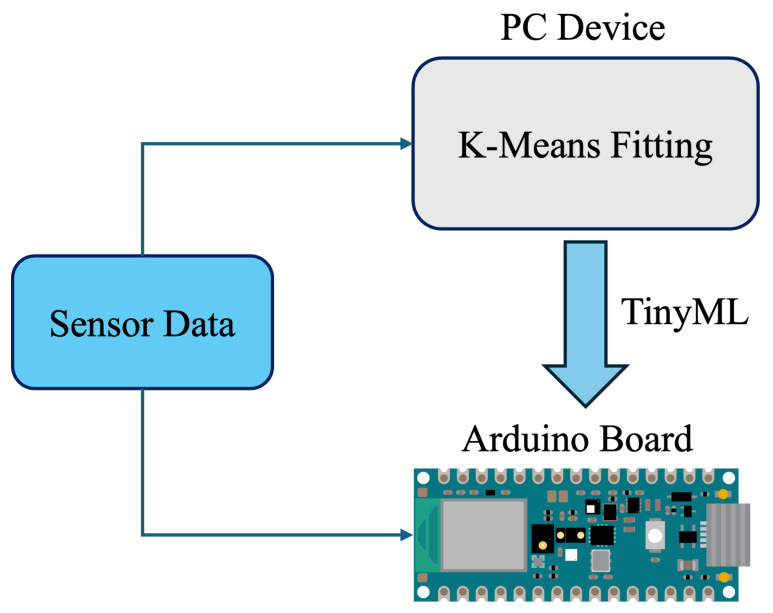
Workflow of the *K*-means implementation on microcontroller.

The implementation of ML algorithms on a microcontroller, known as TinyML [44], focuses on bringing ML capabilities to resource-constrained devices. These devices, with their limited computational power and memory resources, require careful optimization to handle the demands of ML algorithms. Many microcontroller applications require real-time processing, and the iterative nature and high computational demands of these algorithms can lead to delays. These constraints necessitate careful consideration and optimization to make these algorithms feasible for microcontroller-based applications. The *K*-means is characterized by lower memory usage and computational simplicity compared to other unsupervised algorithms [45]. Figure 6 illustrates the proposed approach.

The model was trained offline by applying the *K*-means algorithm to the dataset of real temperature measurements. The resulting centroids were then stored in the microcontroller firmware. Although the clustering was performed in an unsupervised manner, true labels (available for evaluation) were used to compute metrics, verifying the suitability of the model before deployment.

## 4. Performance Evaluation

The aim of the proposed approach is to separate the temperature measurements in different clusters corresponding to a different class of fastening. Each temperature measurement represents a single reading, which is taken to evaluate the health status of the switchgear in a given instant. Indeed, the obtained measurements are employed to learn the three different classes of fastening, corresponding to three health statuses of the MV switchgear.

Furthermore, for comparison purposes, the algorithm of spectral clustering is considered, too. Spectral clustering uses graph theory and linear algebra to reduce the problem to a lower-dimensional space through the data similarity matrix. The similarity matrix is a quantitative evaluation of the relative similarity of each pair of points in the dataset. After the dimension reduction, the clustering technique of *K*-means is applied [40]. Spectral clustering was included in this analysis to explore whether its ability to identify separable clusters could offer an advantage over the more conventional *K*-means approach. Despite its higher computational complexity, it has been shown to perform well in scenarios where data may exhibit global structures or manifold-like geometries, which might not be well captured by centroid-based methods. This approach excels in capturing complex patterns, but it is computationally and memory intensive.

The *K*-means and the spectral clustering are tested on both synthetic data and real measurements derived from the DMS described in Section 3.1. Specifically, the synthetic datasets were generated by varying the number of samples and different values of measurement uncertainty. Since every measurement is inherently affected by uncertainty—due to sensor limitations, environmental conditions, and noise [46,47]—variability in the number of samples should be considered when evaluating algorithm robustness. In this paper, the performance of the clustering algorithms is investigated not only on real-world data but also on uncertainty-augmented synthetic datasets. This approach simulates more realistic operating conditions and helps assess the resilience and generalization capability of the proposed methods in the presence of noise. The key steps in this process include data collection, the generation of synthetic datasets, and evaluation of the algorithms. Finally, the performance of the algorithms was validated using real measurements. This validation also includes the practical implementation on the Arduino board, as described in Section 3.3. To this aim, the clustering algorithms were initially developed and evaluated in Python 3.13.1 using the Scikit-learn library with both real temperature measurements and uncertainty-augmented synthetic datasets collected from the DMS. Subsequently, the *K*-means algorithm was deployed on a microcontroller, using temperature measurements from the DMS. This allowed for a direct comparison between PC-based results and on-device performance, confirming the applicability of the approach for onboard fault detection in resource-constrained environments. To validate model equivalence, the clustering results produced by the *K*-means algorithm on the Arduino board were directly compared to those obtained in Python on the PC, using the same temperature datasets as input. Moreover, fitting and predicting times have been measured for a comprehensive evaluation.

### 4.1. Performance Metrics

While addressing clustering, as concerns performance evaluation, several metrics can be taken into account. Specifically, metrics can be supervised or unsupervised, depending on the availability of data labels. Since in the current evaluation the labels are known, supervised metrics are considered. Thus, both *adjusted rand index* (ARI) and *validity* ($V_{1}$) are chosen. *Silhouette* is then chosen as unsupervised metric.

The ARI metric considers clustering similarity, which is adjusted for the chance grouping of elements:

(6) ARI=∑ijnij2−∑iai2∑jbj2/n20.5∑iai2+∑jbj2−∑iai2∑jbj2/n2,

where $n_{ij}$ is the number of elements in the intersection of clusters *i* and *j*, $a_{i}$ and $b_{j}$ are the sums over the rows and columns of the contingency table, respectively, and *n* is the total number of elements. ARI ranges from −1 to +1, where values closer to 1 indicate a high similarity between clusters, 0 suggests random clustering, and negative values indicate dissimilarity.

The $V_{1}$ metric is based on the two opposite criteria of homogeneity *h*, which occurs when for each cluster, all elements belong to the same class, and completeness *c*, which occurs when for each class, all elements belong to the same cluster. $V_{1}$ is given by the harmonic mean of *h* and *c*, providing a balanced assessment of clustering quality:

(7) $$V_{1}=2\;\cdot \;\frac{h\;\cdot \;c}{h+c}.$$

It ranges from 0 to 1 with higher values indicating better clustering performance.

Finally, the Silhouette metric is defined as follows:

(8) $$Silhouette=\frac{b-a}{\max(a,b)},$$

with *a* representing the average of intra-cluster distances, i.e., the distances between a point and all other points in the same cluster:

(9) $$a=\frac{1}{|C_{i}|-1}\sum_{x^{\prime }\in C_{i},x^{\prime }\neq x}d\left(x,x^{\prime }\right),$$

and *b* representing the smallest average of inter-cluster distances, i.e., the distances between a point and all points in the nearest cluster:

(10) $$b=\min_{j\neq i}\frac{1}{|C_{j}|}\sum_{x^{\prime }\in C_{j}}d\left(x,x^{\prime }\right),$$

where $C_{i}$ and $C_{j}$ are clusters, and $d(x,x^{\prime })$ is the distance between two samples *x* and $x^{\prime }$. It ranges from −1 to +1, where a value close to +1 indicates well-separated clusters, a value close to 0 suggests overlapping clusters or ambiguous boundaries, and a value close to −1 implies that points may be assigned to the wrong cluster.

### 4.2. Numerical Analysis

The experimental data consist of temperature measurements from the thermocouple 1 and the thermocouple 2, categorized by fastening, i.e., with classes 0, 1, and 2, corresponding to 60 Nm, 40 Nm, and 4 Nm, respectively. The 100 measurements acquired for each fastening were adopted, resulting in a total of 600 instances for both thermocouples.

Starting from these measurements, various synthetic datasets are generated. For each thermocouple and each fastening class, the mean of the 100 measurements was computed. New data were subsequently generated for each mean value using a uniform distribution, centered around the mean, with upper and lower bounds, respectively, set to ±1.0% and ±1.5% of the mean value. This choice was made in accordance with the Guide to the Expression of Uncertainty in Measurement (GUM) [48], which recommends the use of a uniform distribution when the probability distribution of the quantity is unknown. The uncertainty values of ±1.0% and ±1.5% were chosen as typical of data acquisition systems in DMSs for temperature monitoring [38]. In order to evaluate the performance of unsupervised learning, the synthetic datasets are used as input data for the clustering algorithms. Particularly, five datasets are considered for each mean value. Each dataset comprises a number of samples N={50,100,500,1000,2000}, which are generated for each thermocouple and each fastening. Note that the listed samples are not the total amount of instances in the dataset. For example, the dataset with N=50 comprises 300 instances: 150 for thermocouple 1 and 150 for thermocouple 2 (i.e., 50 for each fastening class). The variation in the number of generated datasets was devised to explore the impact of data availability on the performance of the algorithms. Instead, the considered uncertainty values allow for a comprehensive evaluation of the performance under different operating conditions. This approach facilitates a deeper understanding of how each algorithm performs in the presence of data variability and provides a robust foundation for selecting the most appropriate algorithm based on the specific requirements of the measurement system. Note that all results correspond to single-run evaluations.

In the first place, the datasets are generated with uncertainty ±1.0%. Clustering tasks are performed using measured values from the thermocouple 1 and thermocouple 2. Table 2 and Table 3, respectively, show the achieved results for thermocouple 1 and 2 data corrupted with ±1.0% uncertainty. Looking at the results for thermocouple 1, *K*-means achieves perfect values of ARI and $V_{1}$, indicating its effectiveness in capturing a data structure with well-separated and compact clusters, as reflected in a high Silhouette value. In contrast, spectral clustering shows lower ARI and Silhouette, highlighting the difficulties in achieving cluster separation. This is particularly noticeable in larger datasets. Similar trends are observed in the results achieved with thermocouple 2. Specifically, *K*-means maintains perfect ARI and $V_{1}$ scores across all sample sizes with consistently high Silhouette values around 0.96. On the other hand, spectral clustering shows degraded performance, with ARI ranging from 0.50 to 0.80 and Silhouette values generally low or even negative, especially for larger datasets, indicating poor cluster separation. In general, *K*-means clustering continues to perform consistently across different dataset sizes, being a robust algorithm. In contrast, spectral clustering exhibits significant variability in its performance metrics.

**Figure 7:**
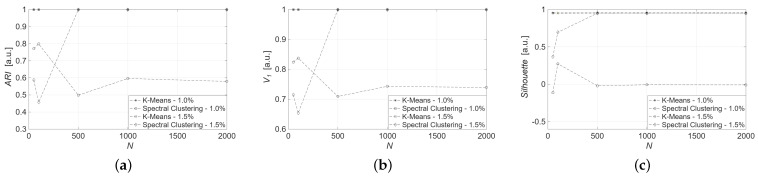
Clustering metrics for synthetic dataset from thermocouple 1: (**a**) ARI, (**b**) V_1, (**c**) Silhouette.

**Figure 8:**
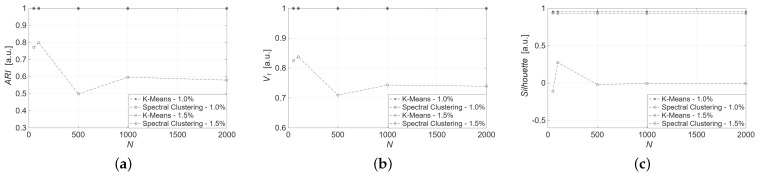
Clustering metrics for synthetic dataset from thermocouple 2: (**a**) ARI, (**b**) V_1, (**c**) Silhouette.

To evaluate the impact of data uncertainty on algorithm performance, the same models were adopted to synthetic datasets generated with uncertainty ±1.5%. Figure 7 and Figure 8 illustrate the metric values for *K*-means and spectral clustering in relation to the applied uncertainty for data from thermocouples 1 and 2, respectively. The results underscore the robustness of the *K*-means algorithm, which consistently achieves high scores for all metrics and data sizes.

In particular, for both sensors and all dataset sizes, *K*-means achieves ARI=1.00, $V_{1}=1.00$, regardless of uncertainty. Spectral clustering shows competitive results only for thermocouple 1, with ARI=1.00, $V_{1}=1.00$, and Silhouette=0.94, considering at least N=500 and ±1.5% uncertainty. When dealing with fewer samples and ±1.0% uncertainty, the performances are not satisfing. Moreover, its performance drops significantly on the smaller dataset of size N=50: ARI=0.59, $V_{1}=0.72$ and Silhouette=0.37, respectively.

This behavior suggests that spectral clustering is sensitive to data sparsity. Nevertheless, *K*-means remains the most robust algorithm, consistently delivering perfect clustering results regardless of uncertainty or sample size, due to its reliance on local distances and straightforward centroid optimization.

### 4.3. Validation on Real Measurements

To validate the proposed approach, the algorithms are tested with the real measurements acquired from the DMS described in Section 3.1. Table 4 reports the achieved ARI, $V_{1}$, and Silhouette with the algorithm applied to real measurements. Only the *K*-means results are reported, as it has better performance compared to the spectral clustering algorithm. The results highlight higher *K*-means performance across different datasets. For the thermocouple 1, it scores a perfect value of 1.00 both in ARI and $V_{1}$, indicating perfect clustering quality. For the thermocouple 2, *K*-means achieves lower values for ARI and $V_{1}$, respectively, equal to 0.98 and 0.97. The *K*-means results are confirmed by the *Silhouette*, which accounts for a moderate degree of separability between the three physical regimes when dealing with thermocouple 2 (0.70 vs. 0.77). This confirms its capability to separate the underlying clusters completely despite the presence of a few borderline points that may otherwise lead to sporadic false alarms or missed fault detections in practice. Overall, *K*-means delivers good performance for clustering with acceptable separation and coherence.

Finally, to rigorously establish the validity of the proposed approach, the *K*-means algorithm is implemented on the Arduino board described in Section 3.3 and applied to the real measurements. This implementation begins with fitting the data on a computer, where the *K*-means algorithm is initially run to determine the centroids. The centroids are then saved and transferred to the Arduino board. On the Arduino board, the algorithm utilizes these predefined centroids to classify new input measurements, specifically temperature readings. For each temperature reading, the Euclidean distance to each centroid is calculated, and the measurement is assigned to the nearest cluster. This process ensures that the classification on the Arduino board is consistent with the results obtained from the computer. The accuracy of the Arduino implementation is confirmed by the fact that the clustering results match exactly with those produced by the computer-based *K*-means algorithm. This demonstrates that the method is robust and reliable even when deployed on the limited hardware of an Arduino board.

Table 5 reports the execution times of the fitting and predicting phases. The results reveal a noticeable difference in execution times between the *K*-means algorithm running on a computer and an Arduino board. On the computer, the fitting phase takes between 18.36 ms and 20.07 ms depending on the thermocouple, while the prediction phase is significantly faster. In contrast, the Arduino prediction times are considerably higher, ranging from 3.36 ms to 4.28 ms. This is attributable to Arduino more limited computational resources. Anyway, the results remain consistent with the execution on computer, demonstrating that the *K*-means algorithm is adaptable to different hardware configurations. The disparity in execution times underscores the trade-offs between computational efficiency and hardware limitations, highlighting the need for optimization when deploying algorithms on resource-constrained devices.

Despite the limited resources of the Arduino board, the implementation requires only a small fraction of the available memory. The code is lightweight, consisting of a simple distance calculation to a set of precomputed centroids and the centroids themselves occupy minimal memory. Both flash and RAM usage remain well below capacity, leaving sufficient room for additional features, such as multi-dimensional inputs or integration with other sensors. This confirms that the proposed method is well suited for deployment on embedded systems.

## 5. Conclusions

This study introduces a distributed, microcontroller-based monitoring system that leverages unsupervised machine learning for the real-time health assessment of MV switchgears under varying thermal conditions. By integrating multiple temperature sensors and implementing a K-means clustering algorithm directly on low-cost microcontrollers, the system achieves reliable fault detection and anomaly identification without the need for labeled data or high-performance computing resources. The approach is particularly well suited for real-world deployment, addressing practical constraints such as limited communication bandwidth, energy efficiency, and the absence of centralized infrastructure. Validation on both synthetic and real-world datasets demonstrates that the proposed method maintains robust clustering performance, even in the presence of noise, and ensures acceptable execution times on resource-constrained devices like an Arduino board. This confirms the feasibility of executing offline-trained models on embedded platforms, enabling local processing and reducing latency by eliminating the need for constant data transmission to external servers. Overall, the solution offers a scalable, cost-effective, and responsive architecture that supports predictive maintenance strategies, contributes to the operational longevity of switchgears, and enhances the overall safety and efficiency of power distribution networks. This work paves the way for further advancements in lightweight AI-driven monitoring systems tailored for constrained environments within the smart grid landscape. Next steps will include the improvement of the solution for a mass scale deployment, including lightweight algorithms, cheap devices, and smart interfaces.

## Figures and Tables

**Table 1:** Initial and final temperature values acquired during the three sessions on site (1) and site (2) through both thermocouples by fastening the upper contact with the current transformer by a torque of 60 Nm, 40 Nm and 4 Nm.

	Fastening	Temperature [°C] on Site (1)		Temperature [°C] on Site (2)	
		Initial	Final	Initial	Final
session 1	60 Nm	47.5	55.0	40.3	46.4
session 2	40 Nm	59.3	66.6	47.0	53.2
session 3	4 Nm	74.5	86.9	57.8	65.2

**Table 2:** Clustering metrics of the algorithms tested on the datasets with uncertainty ±1.0% of thermocouple 1.

Algorithm	Metric	*N*				
		50	100	500	1000	2000
*K*-means	ARI	1.00	1.00	1.00	1.00	1.00
	$V_{1}$	1.00	1.00	1.00	1.00	1.00
	Silhouette	0.97	0.97	0.97	0.97	0.97
spectral clustering	ARI	0.65	0.59	0.63	0.59	0.64
	$V_{1}$	0.77	0.74	0.76	0.74	0.76
	Silhouette	−0.02	0.02	−0.02	0.02	−0.02

**Table 3:** Clustering metrics of the algorithms tested on the datasets with uncertainty ±1.0% of thermocouple 2.

Algorithm	Metric	*N*				
		50	100	500	1000	2000
*K*-means	ARI	1.00	1.00	1.00	1.00	1.00
	$V_{1}$	1.00	1.00	1.00	1.00	1.00
	Silhouette	0.96	0.96	0.96	0.96	0.96
spectral clustering	ARI	0.77	0.80	0.50	0.60	0.58
	$V_{1}$	0.82	0.84	0.71	0.74	0.74
	Silhouette	−0.11	0.27	−0.02	−0.1	−0.01

**Table 4:** Clustering metrics of *K*-means tested on the experimental measurements.

Data	Metric	
thermocouple 1	ARI	1.00
	$V_{1}$	1.00
	Silhouette	0.77
thermocouple 2	ARI	0.98
	$V_{1}$	0.97
	Silhouette	0.70

**Table 5:** Execution times for fitting and predicting phases.

Data	Time [ms]		
	Fitting Phase on Computer	Predicting Phase on Computer	Predicting Phase on Microcontroller
thermocouple 1	20.07	0.82	4.28
thermocouple 2	18.36	0.77	3.36

## Data Availability

The raw data can be made available by the authors on request.
